# Culturally tailored lifestyle interventions for the prevention and management of type 2 diabetes in adults of Black African ancestry: a systematic review of tailoring methods and their effectiveness

**DOI:** 10.1017/S1368980021003682

**Published:** 2022-02

**Authors:** Noor M Wadi, Summor Asantewa-Ampaduh, Carol Rivas, Louise M Goff

**Affiliations:** 1 King’s College London, Department of Nutritional Sciences, Franklin-Wilkins Building, Room 3.87, Waterloo Campus, 150 Stamford Street, London SE1 9NH, UK; 2 Social Research Institute, University College London, London, UK

**Keywords:** Cultural tailoring, Black population, Self-management, Type 2 diabetes, Prevention

## Abstract

**Objective:**

To evaluate the cultural tailoring methods used in type 2 diabetes (T2D), prevention and management interventions for populations of Black African ancestry and to examine their effectiveness on measures of glycaemia.

**Design:**

Three databases were searched in October 2020; eligible studies used a randomised controlled trial (RCT) design to evaluate the effectiveness of culturally tailored lifestyle interventions compared with usual care for the prevention or management of T2D in adults of Black African ancestry. Cultural tailoring methods were evaluated using the Facilitator-Location-Language-Messaging (FiLLM) framework, whereby facilitator refers to delivery by individuals from the target community, language focuses on using native language or language appropriate to literacy levels, location refers to delivery in meaningful settings, and messaging is tailoring with relevant content and modes of delivery.

**Results:**

Sixteen RCT were identified, all from USA. The mean age of participants was 55 years, majority female. Six of fifteen RCT reported significant improvements in glycated haemoglobin (HbA1c) at 6 and 8 months; one, in prediabetes, reported significantly improved fasting plasma glucose. Diabetes knowledge improvement (5/7 studies) was associated with HbA1c improvement. The majority tailored to location (12/16), facilitators (11/16), messaging (9/16) and language (6/16) domains of FiLLM. Those with ethnically matched facilitators and those which tailored to more than one domain showed the greatest HbA1C benefits.

**Conclusion:**

This evidence supports the effectiveness of culturally tailored lifestyle interventions for T2D management in populations of Black African ancestry, with further RCT needed to evaluate interventions for T2D prevention and for communities outside of the USA.

It is estimated that globally 463 million adults are living with diabetes, which represents 9·3 % of the world’s population^(1)^. Type 2 diabetes (T2D) disproportionately affects populations of Black African ancestry^(2,3,4)^. In both the USA and UK, where people of Black African ancestry form significant minority groups, T2D is estimated to be three times more prevalent than in White ethnic groups^(5,6)^. Furthermore, it is projected that the African continent will experience the greatest increase in T2D prevalence over the next two decades^(1)^. Overweight and obesity has a significant association with incident T2D^(4,7)^. Populations of Black African ancestry are most likely out of all ethnic groups to be overweight or obese^(8,9,10)^, making it a main driving force in the difference in diabetes rates amongst these communities compared with White ethnic groups. Diabetes-related complications include kidney failure, non-traumatic lower-limb amputations, blindness among adults, heart disease and stroke and have been associated with worse quality of life^(11)^. In the USA, communities of Black African ancestry not only bear a disproportionate burden of developing T2D but they are also twice as likely to experience diabetes-related blindness, 2·3 times more likely to experience lower limb amputations, 3·5 times more likely to have kidney disease^(8)^ and it is the fourth leading cause of death^(12)^.

The burden of T2D and its associated complications occur as a result of prolonged elevations in blood glucose levels, which often occur as a result of poor self-management^(12)^. The higher prevalence of complications seen in populations of Black African ancestry can be associated with poorer rates of disease management, as demonstrated by a 5-year longitudinal study finding that African-Americans spent fewer days engaging in self-care activities compared with White participants^(13)^. Engaging in self-care behaviours (e.g. self-monitoring blood glucose levels, participating in physical activity) plays an important role in the prevention of T2D risk factors^(14)^ as well as the management of disease and its complications. Diabetes self-management education intervention trials conducted in largely White ethnic groups have been shown to be effective at reducing glycated haemoglobin (HbA1c) and fasting blood glucose for up to 12 months^(15,16)^. However, a 2018 meta-analysis examining diabetes self-management education specifically in populations of Black African ancestry found no effect on HbA1c in these populations^(17)^. This is perhaps due to inadequate matching of interventions to specific cultural barriers and needs such as historical barriers of racism, communication needs (e.g. language/literacy), low socio-economic status, high levels of food insecurity and limited access to safe areas and healthcare^(18)^.

Cultural tailoring of interventions is proposed as a key means by which to overcome these inequalities. There have been several behavioural interventions developed for African-American populations, incorporating culturally tailored strategies showing improvement of CVD risk profiles, smoking cessation, dietary behaviours and weight loss, but diabetes self-management and, in particular, prevention studies have not been as widely studied for these communities^(19)^. The available literature in populations outside of the USA is very scarce. Interventions of these types include a wide variation of culturally tailored strategies, where some may include one or a combination of strategies and others give little attention to defining them. This makes it challenging in the replication of methods and implementation in the public health sector. Lagisetty *et al.* ^(20)^ proposed a novel framework that characterised four key domains (Facilitator, Location, Language and Messaging) of culturally tailored interventions in order to define patterns that contribute to better outcomes.

The aim of this systematic review is to evaluate the methods of cultural tailoring used in lifestyle interventions for T2D prevention or management for populations of Black African ancestry and to examine the effectiveness of such interventions on glycaemic control.

## Methods

### Data sources and study eligibility

Three databases (Medline, Embase and Psychinfo) were searched for articles published from database inception until the search date (October 2020). The full search string is shown in Supplementary Material: search terms (exploded to retrieve related fields) included the following: ‘diabetes prevention’ or ‘management’, ‘Type 2 Diabetes’ and ‘African ancestry’, ‘African-American’, ‘Black’ or ‘Minority Groups’ in combination with ‘Cultural tailoring’ and ‘Randomised controlled trial’ or ‘intervention’. Grey literature databases and sources were not searched.

To be included, studies had to meet the following criteria: (1) adult population (aged ≥18 years) of Black African ancestry, defined as >50 % of study participants being of Black African ancestry; (2) population diagnosed with T2D or prediabetes, impaired fasting glucose or impaired glucose tolerance; (3) evaluation of a culturally tailored lifestyle intervention (interventions were considered culturally tailored if indicated in text as being culturally tailored, culturally adapted or cultural sensitivity tailoring); (4) intervention outcome measures included change in HbA1c or fasting blood/plasma glucose and (5) were randomised controlled trials. Studies were not excluded on the basis of language.

### Study selection and data extraction

Titles and abstracts were screened by two independent assessors (NW, SA) and excluded if they did not meet the eligibility criteria, where eligibility could not be ascertained by title and abstract, full texts were reviewed by one assessor (NW). Full texts were reviewed to ensure eligibility by one author and in the case of uncertainty, a second independent reviewer (LG) was consulted to make the final decision. Additional studies were identified by hand searches of bibliographies and reference lists.

Descriptive data were extracted by one assessor (NW) to include year of publication, study design, number of participants, age, gender, ethnicity of targeted population, presenting condition (diabetes or pre-diabetes), setting of intervention, duration of intervention, intervention characteristics and control-arm characteristics. Outcome data extracted included mean/median HbA1c/fasting glucose, mean change (or baseline and post-intervention if not included) and statistical significance. If available, mean weight (in kg, or BMI in kg/m^2^) and weight change were also extracted, as well as measurements of diabetes knowledge as a secondary outcome. Details of components of the cultural tailoring of the intervention were extracted in accordance with the Facilitator-Location-Language-Messaging domains proposed by Lagisetty *et al.* ^(20)^ This was used to classify culturally tailored components based on use of Facilitators, Location, Language and Messaging. Information on Facilitator was regarded as the use of ethnically matched community health workers (CHW), community-based facilitators or healthcare professionals. Location of intervention referred to the setting where the intervention took place. The language domain accounted for adjustments in intervention to match literacy or language of the population, and messaging content encompassed altered content based on cultural specifics to faith, family, gender or diet.

### Risk of bias

Risk of bias was assessed independently by two reviewers (NW, LG) using the Cochrane tool for assessing risk of bias in RCT^(21)^, adjusting the tool for lifestyle interventions. Bias was assessed at the outcome level based on elements including randomisation of sequencing, allocation concealment, blinding of outcome assessment and incomplete outcome data and given a rating of present (+), not present (-) or unclear (?) which was used to determine overall risk of individual studies.

## Results

**Fig. 1:**
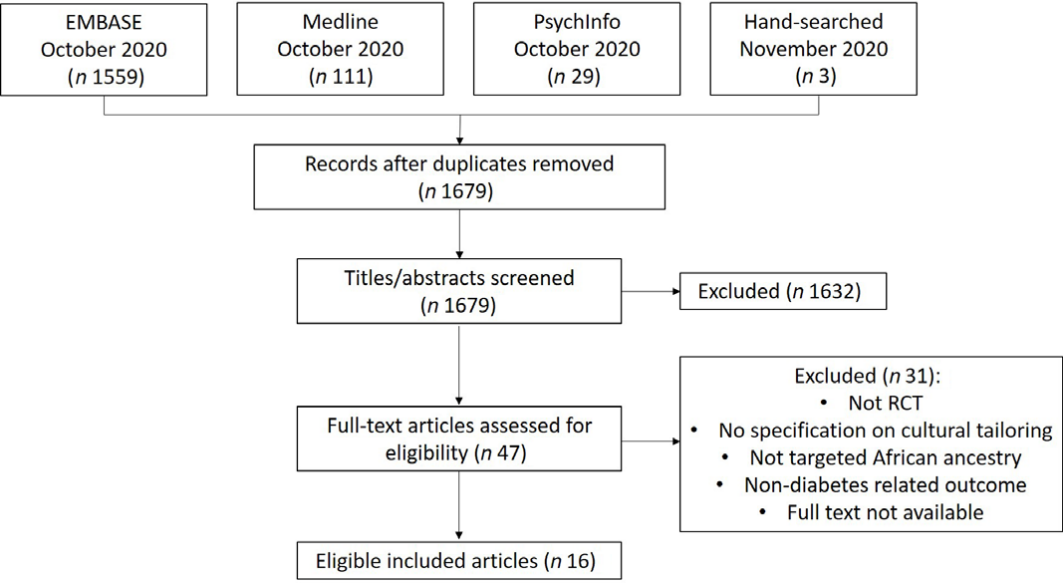
PRISMA flow diagram

A total of 1702 citations were retrieved from EMBASE, MEDLINE, PsycINFO and hand searches of reference lists (Fig. 1). After removing duplicates, 1679 abstracts remained. Following screening of titles and abstracts, forty-seven full-text articles were further screened for inclusion. In total, sixteen RCT were eligible for inclusion.

Table 1 displays the characteristics of the included RCT; all took place in the USA with a total of 3568 participants. Mean age of participants was 54·8 years, and all studies recruited more than 50 % women; four studies included only women^(22,23,24,25)^. Eleven of the studies exclusively enrolled adults of Black African ancestry and five targeted multiple ethnicities with more than 50 % of the population group being of Black African ancestry^(26,27,28,29,30)^. Two studies enrolled adults with pre-diabetes or at high risk of T2D^(26,31)^, and seven reported HbA1c as a secondary outcome, with primary outcomes being weight loss (*n* 5)^(26,29,31,32,33)^, physical activity levels^(25)^ and 3-year hospital event^(34)^. There was one pilot RCT^(32)^. Follow-up ranged from 12 weeks^(23,31)^ to 20 weeks^(33)^, 6 months^(28,32)^, 12 months^(19,24,25,26,27,29,35)^, 18 months^(36)^ and 24 months^(22,30,34)^. Nine provided group sessions^(19,22,23,26,31,32,33,35,36)^, and five individual^(24,27,29,30,34)^ and two used a mixed approach of both individual and group sessions^(25,28)^.

**Table 1:** Study characteristics

Author, year, setting	Participants	Intervention/control	Population	Mean age	95 % CI	sd (years)	Intervention	Control	Duration	Outcomes
Pre-diabetes										
Ackerman *et al.* 2015 USA	509	I: 257; C: 252	57 % African American	51·0		12·1	16 face to face small group session delivered over 16 to 24 weeks followed by monthly support meetings.	Standard care	24 weeks	Primary: Body weight Secondary: HbA1c
Sattin *et al.* 2016 USA	604	I: 317; C: 287	African American	46·6		10·9	12 weekly group sessions of faith-based adaptation of Diabetes prevention programme. 6 booster sessions included post-baseline.	12 weekly group sessions of standard health education, non-diabetes specific.	12 weeks	Primary: Weight change Secondary: FPG
Type 2 diabetes										
Melkus *et al.* 2010 USA	109	I: 52; C: 57	African American Women	48·0		10·0	11-week culturally relevant group DSMT, coping skills training and diabetes care intervention	10-week group-based usual diabetes education and diabetes discussion	12 weeks	HbA1c
Murrock *et al.* 2009 USA	46	I: 24; C: 22	African American Women	62·8		10·1	12 weekly 60-minute peer supported dance class	10-week usual diabetes education	12 weeks	HbA1c
Lutes *et al.* 2012 USA	200	I: 100; C: 100	African American Women	53·5		10·2	16 phone-based lifestyle intervention sessions	16 educational mailing materials.	12 months	HbA1c
Keyserling *et al.* 2002 USA	200	I: 67; C: 66 & 67	African American Women	59·1			Month 1–6: Traditional Individual clinic counseling visits and two community-based group sessions with monthly phone calls followed. Month 6–12: monthly phone calls and 1 community-based group session.	Clinic intervention only Or mailing educational pamphlets.	16 weeks	Primary: PA Secondary: HbA1c
Samuel Hodge *et al.* 2009 USA	201	I: 117; C: 84	African American	59·1		1·1	12 biweekly Group-based church intervention, 1 individual counseling session and monthly phone contact.	Standard educational pamphlets by mail and 3 bimonthly newsletters	12 months	HbA1c
Lynch *et al.* 2014 USA	61	I: 26; C: 29	African American	54·1		10·0	18 Weekly Group-based diabetes self-management class and weekly telephone calls.	Short-term diabetes care: 2 group based self-management training.	6 months	Primary: Weight loss Secondary: HbA1c
Lynch *et al.* 2019 USA	211	I: 106; C: 105	African American	55·0		10·3	28 group sessions over 12 months; weekly (months 1–4); biweekly (months 5–8); monthly (months 9–12) in a community setting.	2 Standard DSME sessions in clinic	12 months	HbA1c
Anderson *et al.* 2005 USA	239	I: 125; C: 114	African American	61·0		11·4	6 weekly group session of culturally specific ‘me	6-week delay.	6 weeks	HbA1c
Gary *et al.* 2017 USA	542	I: 269; C: 273	African American	58·0		11·0	Individual tailored efforts by nurse and CHW, minimum 3x per year.	Telephone calls and mailing of standard information brochures every 6 months.	24 months	Primary: 3-year hospital events Secondary: HbA1c
Samuel-Hodge *et al.* 2017 USA	108 (54 dyads)	I: 72; C: 36	African American	51·0			20 weekly-group based diabetes education of family dyads.	Delayed intervention	20 weeks	Primary: Weight loss Secondary: HbA1c
Ruggiero *et al.* 2014 USA	266	I: 134; C: 132	52·6 % African American (+Latin American)	53·2		12·4	TAU AND Medical assistant self-care coaching involving face-to-face coaching delivered quarterly at routine checkups and monthly telephone follow-ups.	TAU AND “Diabetes: youre in control booklet” (adapted for low literacy)	12 months	HbA1c
Spencer *et al.* 2011 USA	164 (94 AA)	I: 72; C: 92	African American (53 %) and Latino	52·5	50, 54·5		CHW conducted 11 2-h group sessions on diabetes education every 2 weeks at community locations. 2 home visits per month and 1 clinic visit with participant.	6-month delayed intervention.	6 months	HbA1c
Mayer-Davis *et al.* 2004 USA	152	I: 49; C: 47 & 56	82 % African American	60·3		8·6	16 weekly individual meeting with nutritionist.	2 control arms; RI: Condensed version; 4 1-h sessions (3 group; 1 individual) UC: 1 individual session	12 months	Primary: Weight loss Secondary: HbA1c
Sharp *et al.* 2018 USA	244	I: 120; C: 124	73 % African American	54·2		11·2	Monthly CHW for first 3–4 months; accompanied them to PCP and pharmacist encounters	Pharmacist intervention providing medication and disease management	24 months	HbA1c

HbA1c, haemoglobin A1c; FPG, fasting plasma glucose; DSMT, diabetes self-management training; PA, physical activity; DSME, diabetes self-management education; CHW, community health worker; TAU, treatment as usual; AA, African American; RI, reimbursable lifestyle intervention; UC, usual care; PCP, primary care physician.

All interventions were described as culturally tailored/adapted for African-American communities. Details of the cultural tailoring methods are provided in Table 2 and described in detail below.

**Table 2:** Facilitator-Location-Language-Messaging domains included in cultural tailoring

Study author and year	Facilitators (F)	Location (Lo)	Language (La)	Message (M)	Other (O)
Ackerman *et al.* 2015	YDPP instructors	Local community or YDPP	–	–	Offered free access to gym
Sattin *et al.* 2016	Church health advisors	Local church	–	–	–
Melkus *et al.* 2010	Nurse	–	–	Diet, beliefs, videos	Transportation, parking and/or childcare
Murrock *et al.* 2009	AA dance instructor	Community based outpatient clinic	–	Gospel music; faith	–
Lutes *et al.* 2012	CHW	–	–	–	Phone based; given tools (scale, glucose monitor and pedometer)
Keyserling *et al.* 2002	Community diabetes advisor	Community health centres	–	–	–
Samuel-Hodge *et al.* 2009	Peer counselor	Local church	Literacy	Opened with prayer; faith	–
Lynch *et al.* 2014	RD; 2 AA peer supporters	Local city park	Literacy	Diet	
Lynch *et al.* 2019	RD; peer leader	Community setting	Literacy	Faith, diet	–
Anderson *et al.* 2005	RD or nurse	Community based locations	Literacy of educational materials	Ethnic recipes; diet	–
Gary *et al.* 2017	CHW	Community centres; Home; Clinics	CHW assistance with forms	–	–
Samuel-Hodge *et al.* 2017	RD	–	–	Family	–
Ruggiero *et al.* 2014	Medical assistant coaches	Primary care in underserved community	Medical Coach assistance	Educational material: Diet	–
Spencer *et al.* 2011	CHW	Home & community locations	–	–	Access to health facilities
Mayer-Davis *et al.* 2004	Nutritionist	–	–	Diet	–
Sharp *et al.* 2018	CHW Pharmacist	Home visits	–	–	

YDPP, YMCA diabetes practitioner; AA, African American; CHW, community health worker; RD, registered dietitian.

### Effectiveness

Fifteen studies measured HbA1c (%) as an outcome, the remaining study by Sattin *et al.* ^(31)^ on diabetes prevention measured fasting plasma glucose. Of the fifteen HbA1c studies, six reported significant differences (*P*-value of <0·05) in HbA1c at follow-up favouring the intervention^(19,23,27,28,33,36)^. A further five reported HbA1c changes favouring the intervention group but were NS (*P* > 0·05)^(22,24,29,32,34,35,36)^. Two reported outcomes favouring the control (*P* = 0·33; *P* = 0·73, respectively)^(19,25)^. Two studies targeted diabetes prevention, Sattin *et al.* ^(31)^ measured fasting plasma glucose as a secondary outcome and found significant improvement in fasting plasma glucose in pre-diabetic participants in the intervention group compared with the control (-10·93 *v*. + 4·22 mg/dl, *P* = 0·017). In contrast, Ackermann *et al.* ^(26)^ measured change in HbA1c as a secondary outcome and found no significant difference between groups at follow-up (*P* > 0·05).

This review also considered diabetes knowledge as an indicator of intervention effectiveness. Seven of the included studies reported on diabetes knowledge using a range of questionnaires including (a) Diabetes Knowledge Test^(22)^; (b) Diabetes Knowledge Scale^(19,25)^; (c) Nutrition Knowledge Test^(32,36)^; (d) perceived understanding of diabetes and (e) Spoken Knowledge in Low Literacy Diabetes Scale^(30)^. Five studies reported statistically significant improvements in knowledge compared with the control group (*P* = 0·003; *P* = 0·037; *P* = 0·010; *P* < 0·001; *P* = 0·048, respectively)^(19,25,32,35,36)^.

Twelve out of the sixteen articles reported on weight loss as either a primary^(26,29,31,32,33)^ or secondary outcome. Five studies reported a statistically significant reduction in weight, favouring the intervention (*P* = 0·046; *P* < 0·001; *P* < 0·010; *P* = 0·001; *P* < 0·0001, respectively)^(24,26,29,31,33)^, whilst seven found no significant difference between intervention and control groups (*P* > 0·05).

### Methods of cultural tailoring – Facilitator-Location-Language-Messaging framework

#### Facilitators

Eleven of the sixteen interventions were delivered by, or supported by, members of the community. These members were all ethnically concordant and included a dance instructor^(23)^, community diabetes advisor^(19,25)^, community health worker (CHW)^(24,28,30,34)^, peer leaders^(32,36)^, church health advisors^(31)^ and YMCA instructors^(26)^. Five interventions used healthcare professionals who consisted of registered dietitians^(33,35)^, nutritionist^(29)^, medical assistant coach^(27)^ or a nurse^(22)^ Three studies used a combination of community members and healthcare professionals^(30,32,36)^. No specification was given regarding ethnic matching of healthcare professionals except for in Ruggiero *et al.* ^(27)^, where the medical assistant coach was ethnically matched to the participants. Of the eleven studies using members of the community, four reported statistically significant reduction in HbA1c at 8 months (*P* = 0·009), 12 weeks (*P* = 0·020) and 6 months (*P* < 0·010; *P* = 0·030, respectively)^(19,23,28,36)^; however, two of these showed no significant difference (*P* = 0·330; *P* = 0·520, respectively) at 12-month follow-up^(19,36)^. A further three showed outcomes favouring the intervention but were NS (*P* > 0·05)^(24,32,34)^.

#### Location

Almost all studies (12/16) used convenient locations in the participants’ communities including community centres (*n* 7)^(23,25,26,28,34,35,36)^, local churches (*n* 2)^(19,31)^, local park (*n* 1)^(32)^ or a local primary care centre specified as being in an underserved community (*n* 2)^(27,30)^. Three of these also included home visits^(28,30,34)^. In total, six of the twelve studies found the intervention resulted in a statistically significant difference between groups, including 3/7 studies using community centres^(23,28,36)^. Both studies using local churches found significant differences between groups, favouring the intervention^(19,31)^. In the two studies conducted in primary care centres, both favoured interventions, but only one was significant^(27)^ (*P* < 0·001).

#### Language

Four studies adapted educational materials for literacy^(19,32,35,36)^, of which two found significant improvements in HbA1c favouring the intervention at 6 months^(36)^ and 8 months^(19)^ but not at 12 months. Two additional studies used CHW or medical staff to assist with forms and understanding to increase comprehension^(27,34)^. Of these, only Ruggiero *et al.* ^(27)^ found a significant improvement in outcome measures between groups.

#### Messaging

The messaging component consisted of altering the content of the interventions and were divided into four subcategories: diet, faith, family and gender. Six tailored their intervention content to diet (Table 3)^(22,27,29,32,35,36)^. Half specifically provided tailored cookbooks^(22,29,35)^ and others (*n* 3) included cultural tailoring of diabetes nutritional education^(27,32,36)^. These three all showed statistically significant improvements in HbA1c in the intervention group compared with the control group. Four studies targeted their content based on faith^(19,22,23,36)^; three of them were successful in improving glycaemic control in the intervention compared to control group. Only one study targeted content to family by use of family dyads^(33)^ and found a significant improvement in intervention group compared with the control.

**Table 3:** Effect of culturally tailored interventions on HbA1c, weight and diabetes knowledge

Study author, year	HbA1c/FPG		*P* value between group	Weight		*P* value between group	Diabetes knowledge		*P* value between group
	Intervention	Control		Intervention	Control		Intervention	Control	
Ackerman *et al.* 2015	Baseline: 6·1 % Follow-up: NR	Baseline: 6·0 % Follow-up: NR	NS	Baseline: 103·0 kg 12-month follow-up: −2·5 kg	Baseline: 101·7 kg 12-month follow-up: −0·3 kg	*P* < 0·001	NR	NR	
Sattin *et al.* 2016	FPG Baseline: 90·1 ± 10·0 mg/dl Change at 12 weeks: −1·85 mg/dl	FPG Baseline: 89·9 ± 9·4 mg/dl Change at 12 weeks: −0·06 mg/dl	*P* = 0·486	Baseline: 98·4 kg Mean change 12 weeks: −2·6 kg	Baseline: 99·0 kg Mean change 12 weeks: −0·50 kg	*P* = 0·001	NR	NR	
Melkus *et al.* 2010	Baseline: 8·0 % 3-month follow-up: 7·3 %* 24-month follow-up: 7·4 %*	Baseline: 8·3 % 3-month follow-up: 7·4 %* 24-month follow-up: 8·0 %	NS	NR	NR		Baseline: 82† Follow-up: NR**	Baseline: 79† Follow-up: NR**	NR
Murrock *et al.* 2009	Mean change: −0·5 %*	Mean change: +0·3 %	*P* = 0·020	Mean change (lb): −4·9*	Mean change (lb): −3·7*	NS	–	–	–
Lutes *et al.* 2012	Baseline: 9·1 % Mean change: −0·29 %	Baseline: 9·0 % Mean change: +0·05 %	*P* = 0·789	Baseline: 98·1 kg Mean change: −1·35 kg	Baseline: 104·2 kg Mean change: −0·39 kg	*P* = 0·046	NR	NR	–
Keyserling *et al.* 2002	Baseline: 10·7 % 6-month follow-up: 10·7 % 12-month follow-up: 10·8 %	Baseline (B): 11·1 % (C): 11·3 % 6-month follow-up: (B): 11·1 % (C): 11·5 % 12-month follow-up: (B): 10·9 % (C) 10·7 %	*P* = 0·730	Baseline: 207 lb 6-month follow-up: 207 lb 12-month follow-up: 212 lb	Baseline (B): 202 lb (C): 210 lb 6-month follow-up: (B) 202 lb (C) 210 lb 12-month follow-up: (B) 208 lb (C) 212 lb	*P* = 0·520	Baseline: 9·0‡ 6-month follow- up: 10·5 12-month follow-up: 10·7	Baseline‡(B): 8·6 (C): 9·4 6-month follow-up: (B): 9·9 (C): 9·6 12-month follow-up: (B) 9·8 (C): 10·1	*P* = 0·037
Samuel Hodge *et al.* 2009	Baseline: 7·7 % Adjusted 8-month follow-up: 7·4 % 12-month adjusted follow-up: 7·5 %	Baseline: 7·9 % Adjusted 8-month follow-up: 7·8 % 12-month adjusted follow-up: 7·6 %	8 months *P* = 0·009 12mo *P* = 0·330	Baseline: 99·0 kg 8-month follow-up: 98·0 kg 12-month follow-up: 97·0 kg	Baseline: 95·0 kg 8-month follow-up: 98·0 kg 12-month follow-up: 97·0 kg	8-month: *P* = 0·940 12 months: *P* = 0·710	Baseline: 8·9‡ 8-month follow-up: 10·7	Baseline: 8·4‡8-month follow-up: 9·8	*P* = 0·003
Lynch *et al.* 2014	Intervention: Baseline: 7·9 % Mean change: −0·5 %*	Control: Baseline: 7·7 % Mean change: 0·1 %	*P* = 0·100	Baseline: 215·9 lb Mean change: −2·8 kg*	Baseline: 219·4 lb Mean change: −1·1 kg	*p* = 0·170	Baseline††,§: 57·0 Mean change: 18·0	Baseline: 63·0 Mean change: 7·6	*P* = 0·010
Lynch *et al.* 2019	Median Baseline: 8·6 % Mean change 6 months: −0·76 12 months: −0·63 18 months: −0·58	Median baseline: 8·4 % Mean change 6 months: −0·21 12 months: −0·45 18 months: −0·33	6 months: *P* = 0·030 12 months: *P* = 0·520 18 months: NS	NR	NR		Baseline‡: 34·0 Change at 12 months: 11·1* 18 months: 7·7	Baseline: 35·0 Change at 12 months: 6·0 18 months: 4·5	12 months: *P* = 0·002 18 months: *P* = 0·048
Anderson *et al.* 2005	Baseline: 8·7 % 6 weeks: 8·3 %**	Baseline: 8·4 % 6 weeks: 8·1 %**	NS	Baseline: 201·2 lb 6-week follow-up: 199·7 lb*	Baseline: 201·2 lb 6-week follow-up: 201·4 lb	NS	Baseline: ‖ 2·7 6-week follow-up: 3·4**	Baseline: 2·6 6-week follow-up: 2·8	*P* < 0·001
Gary *et al.* 2017	Baseline: 7·9 % 24-month follow-up: 7·7 %	Baseline: 8·0 % 24-month follow-up: 7·9 %	NS	NR	NR		NR	NR	
Samuel Hodge *et al.* 2017	Baseline: 7·5 % Adjusted mean change: −0·51 % Unadjusted mean change: −0·45 %	Baseline: 7·6 % Adjusted mean change: 0·38 Unadjusted mean change: 0·26 %	*p* = 0·040	Baseline: 105·4 kg Adjusted mean change: −4·3 kg Unadjusted mean change: −4·4 kg	Baseline: 107·1 kg Adjusted Mean change: +1·4 kg Unadjusted mean change: +1·6 kg	*P* < 0·0001	NR	NR	
Ruggiero *et al*. 2014	Baseline: 9·1 % Follow-up: n/a	Baseline: 8·3 % Follow-up: n/a	Unadjusted group effects: *P* < 0·001	NR	NR		NR	NR	
Spencer *et al*. 2011	Adjusted baseline: 8·6 % Adjusted 6-month follow-up: 7·8 %**	Baseline: 8·5 % 6-month follow-up: 8·5 %	*P* < 0·010	Baseline: 32·7 kg/m^2^ 6-month follow-up: 33·0 kg/m^2^	Baseline: 34·1 kg/m^2^ 6-month follow-up: 33·7 kg/m^2^	NS	NR	NR	
Mayer-Davis *et al*. 2004	Baseline: 10·2 % Mean change: 6 months −1·6 %	Baseline: RI: 9·7 % UC: 9·6 % Mean change: 6 months: RI: −0·80 % UC: −1·1 %	NS	Baseline: 99·5 kg Mean change 6 months: −2·2 kg** 12 months: −2·2 kg*	Baseline: (RI) 100·0 kg (UC) : 93·0 kg Mean change: 6 months: (RI): −1·1 kg (UC): −0·40 kg 12 months: (RI): −0·8 kg (UC): −0·2 kg	6 months: *P* < 0·010 between usual care	NR	NR	
Sharp *et al.* 2018	Baseline: 9·4 % Mean change: −0·45 %	Baseline: 9·6 % Mean change: −0·43 %	NS	Baseline: 35·7 kg/m^2^ Mean change: −0·20 kg/m^2^	Baseline: 36·8 kg/m^2^ Mean change: −0·22 kg/m^2^	NS	Baseline:¶ 5·3	Baseline¶ 5·4	NS

HbA1c, haemoglobin A1C; FPG, fasting plasma glucose; NR, not reported; NS, no significance; RI, reimbursable lifestyle intervention; UC, usual care.

* Significant within group difference; *P* < 0·05.

† The Diabetes Knowledge Test; a 25-item self-administered multiple- choice objective test developed by D’Eramo-Melkus, Wylie-Rosett, and Hagan (1992).

‡ 16-item adaptation of the Diabetes Knowledge Scale.

§ Adapted version of the Nutrition Knowledge Questionnaire; Score range 0–62. Higher scores indicate greater nutrition knowledge.

‖ Perceived understanding of diabetes; sale 1 = poor, 5 = excellent.

¶ Spoken Knowledge in Low Literacy in Diabetes scale.

** Significant within group difference; *P* < 0·001.

†† Mean % of correct answers.

Other methods were used in four studies to increase acceptability of the intervention. D’Eramo Melkus *et al.* ^(22)^ provided free transportation, parking and/or childcare to participants and found improvements favouring the intervention at 24 months, although these were NS. Two studies provided free access to health facilities, one finding a significant difference between groups^(28)^ and the other finding no improvement^(26)^. Lutes *et al.* ^(24)^ provided participants with tools for weight, glucose and physical activity monitoring found to favour the intervention, yet results were NS at 12 months (*P* = 0·789).

One of the four studies using only one domain of cultural tailoring found significant HbA1c improvement (*P* = 0·040)^(33)^. Two of the five studies using two domains found significant difference between groups (*P* < 0·010; *P* = 0·017)^(28,31)^. Seven studies incorporated more than two domains, and four found significant difference between groups (*P* = 0·009; *P* = 0·020; *P* < 0·001; *P* = 0·030, respectively)^(19,23,27,36)^ and the remaining showed non-significant improvements favouring the intervention (*P* > 0·05)^(32,34,35)^.

### Risk of bias

**Fig. 2:**
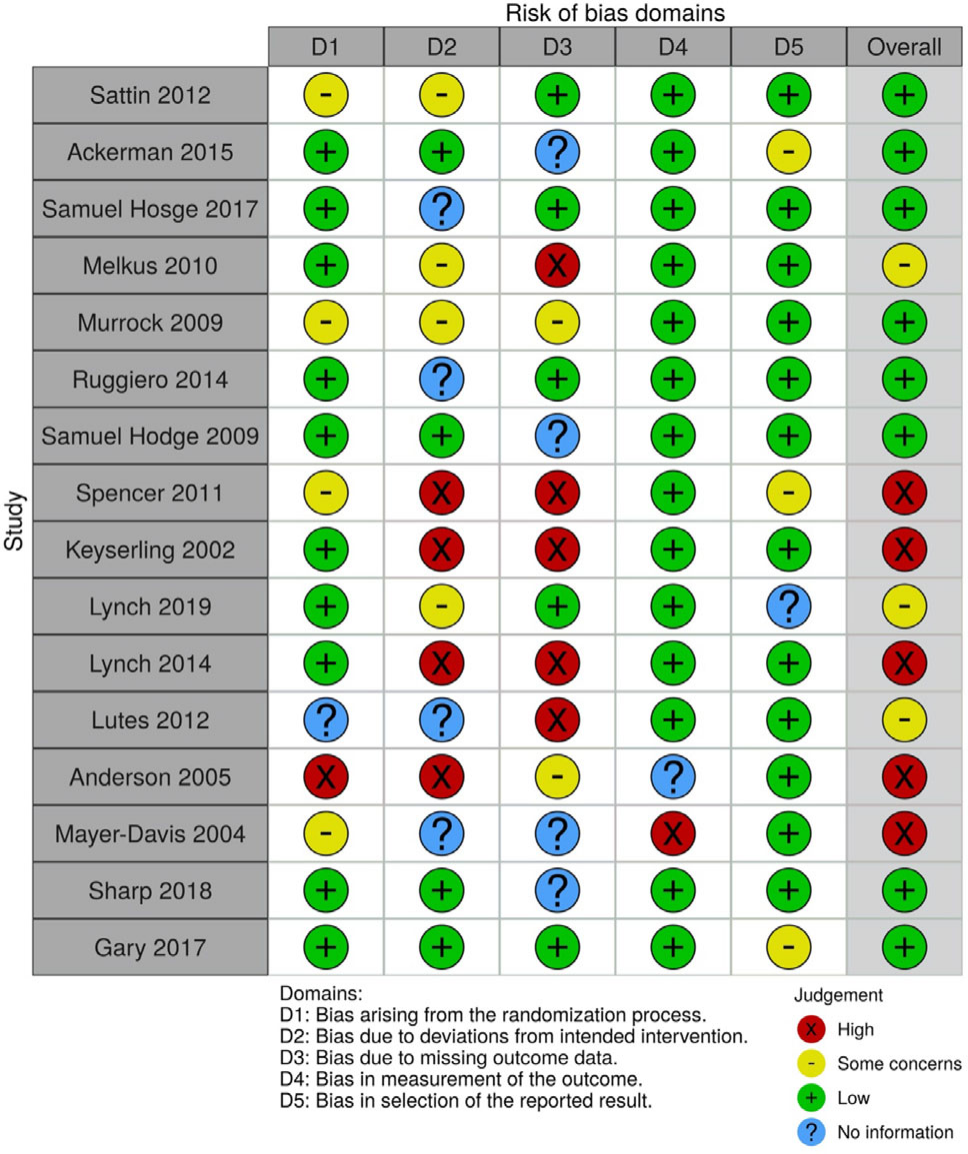
Risk of bias tool

For the majority of studies included in this review, risk of bias was low. The majority of criteria for individual studies was assessed at low to uncertain risk of bias. Due to some studies’ selection bias, bias related to allocation concealment and bias in measurements of outcomes, the quality of the evidence was graded as moderate-low (Fig. 2).

## Discussion

This review systematically investigated the effectiveness of culturally tailored lifestyle interventions on HbA1C and fasting glucose in people with T2D or prediabetes of Black African ancestry. Overall, the use of cultural tailoring resulted in improvements in glycaemic control. These results corroborate the Cochrane review by Attridge *et al.* ^(37)^ who found that culturally appropriate health education improved glycaemic control in participants from ethnic minority communities compared with those receiving usual care. However, the Cochrane review^(37)^ focused on all minority ethnic groups, whereas this review focused solely on populations of Black African ancestry. In contrast to Attridge *et al.* (2014) who reported sustained improvement up to 24 months of follow-up, this review found short-term improvement prevalent up to 8 months^(19)^, but these effects on glycaemic control did not persist significantly at 12 or 24 months. A recent systematic review reported findings that the use of culturally tailored interventions for chronic disease management contributed to improvement in all healthcare outcomes, but that overall results were mixed^(38)^. This outcome was seen in our study, where six articles reported non-significant improvement in HbA1c for the intervention compared with the control. The lack of significance can be explained by HbA1C as secondary outcome, no mention of statistical power or reporting of insufficient sample population to detect power hindering interpretation of true effectiveness of the intervention.

It has been reported that modest weight loss (˜5 %) can improve glycaemic control in T2D patients^(39)^. In the current review, twelve studies reported on weight loss, five as a primary outcome and only three of these found significant improvement^(24,26,29)^. However, weight loss was not associated with improved HbA1C in this population group. The average weight loss noted was 2·5 kg over 12 months, which may not have clinical significance on outcome measures. It is worth considering that greater reductions in HbA1c are seen with higher baseline HbA1c, and Ackermann *et al.* ^(26)^ investigated a pre-diabetic population with low baseline HbA1c (6·1 %), which may explain the insignificance on glycaemic control^(40)^. Using a more sensitive marker of glycaemic control, such as plasma glucose concentrations, may exhibit improvements in pre-diabetic populations^(41)^. The study by Mayer-Davis *et al.* ^(29)^ reported a significant reduction in HbA1c in the control group despite a weight loss of only 0·2 kg. This may in part be due to the introduction of a diabetes management initiative at the clinic during the trial leading to improvements in diabetes self-management. In addition, participant motivation to engage in a 1-year long intervention for self-management improvement, described as the central concept in Wagner’s chronic illness care model^(42)^, may have also played a role in glycaemic improvement.

In contrast, improvements in diabetes knowledge scores were associated with improvement in glycaemic control. Five of the seven studies reporting on diabetes knowledge found significant improvements in intervention knowledge scores, which in the majority of studies also saw a positive impact on HbA1C. Self-management interventions are underpinned by patient education to subsequently increase patient knowledge about a disease^(43)^. The enhancement of knowledge is paramount to facilitate self-directed behaviour change and, consequently, improve health outcomes^(44)^. A retrospective observational study on in-patient adults with T2D reported higher average HbA1C in those who had not received previous diabetes education^(45)^. Further literature supports this association between metabolic control and participation in diabetes education interventions^(46)^. Although there is growing evidence to support this claim, it is important to note that knowledge in isolation has limited impact on behaviour change^(32,47)^. Other factors such as patient attitudes, motivations and the integration of education with other therapies reflect the impact of intervention on behaviour change^(48)^. Culturally tailored interventions are based on the reframing of interventions to match pre-existing beliefs of ethnic minorities to facilitate the learning process^(36)^. The incorporation of these into the interventions in this review is the perceived mechanism for the positive association seen between knowledge and metabolic control. A 2012 pilot observational study found that combining culturally tailored education with shared decision making was a promising strategy for improvement in health outcomes in African-Americans^(49)^.

Resnicow *et al.* ^(50)^ proposed cultural sensitivity as defined by two dimensions; surface structures, involving matching of intervention materials and messages to surface or ‘superficial’ characteristics of a target population to increase acceptance, for example, language; or deep structures, involving incorporation of the cultural, social, historical or environmental forces that target health behaviour in the target population that determines efficacy of the programme. In the current review, the majority of studies tailored to location and facilitators with lesser amount tailoring to messaging and language. Overall, studies that tailored to all four domains showed greater success than those only tailoring to one. Lagisetty *et al.* ^(20)^ reported similar findings indicating the effectiveness of using more than one component of both surface and deep structures of cultural tailoring to increase efficacy of interventions.

This review reported improvements in glycaemic control in a majority of the studies with interventions using members of the community as facilitators. This contributes to the growing body of literature that suggests community members can have a positive impact on improving ethnic minority participants health status^(51)^. Ethnic minorities in the USA and UK are more likely than majority White populations to have lower levels of trust and satisfaction with their physician^(52)^, with African-Americans reportedly preferring a culturally concordant physician^(53)^. This was evidenced in the study by Ruggiero *et al.* ^(27)^ which included the use of a medical assistant coach that was matched to patient’s ethnicity in routine diabetes clinic and found a significant improvement in HbA1c post intervention. This preference may be related to the perceived levels of racism within healthcare systems influencing cultural mistrust^(54)^. Interestingly, fewer than 40 % of primary care clinicians recognise the presence of disparities in health stemming from societal racism^(55)^ presenting a barrier for improved healthcare in ethnic minorities. An RCT of cultural competence training in USA primary care teams demonstrated an increase in clinician awareness of these disparities but reported limited evidence on the clinical impact in African-Americans, perhaps due to the population group being made of predominately Whites (64 %) *v*. 36 % African-Americans^(55)^. Nonetheless, as patient race may influence clinical decisions, more research and attention needs to be given to understandings of race *v*. ancestry or ethnic minority as concepts and to appreciate the need for improving cultural competence of healthcare professionals matched to ancestry to improve health outcomes and increase trust in healthcare systems. The ability of community members to promote health messages to their respective ethnic, cultural or geographical communities gives them the unique capability to bridge the relationship of mistrust between healthcare teams. Patients seemingly have more trust in CHW who understand their socio-cultural barriers, provide social support and can increase relevancy of disease management. A systematic review of strategies to improve response to cultural interventions in T2D by Glazier *et al.* ^(56)^ showed that more successful interventions used a community educator, correlating well with this review. This review found that studies often used CHW who had or lived with someone who had T2D, increasing the relevance and empathy between participants and facilitators.

This review also showed a favouring towards interventions using group sessions for education (*n* 8). In addition to ethnically matched facilitators, group settings elicited feelings of community in some studies. In Murrock *et al.* ^(23)^, the women in the focus group reported feeling ‘disappointed’ when they had to miss a session with other comments including ‘we all had the same thing so you don’t really think about it’ and ‘the fact that it was all different sizes, shapes, ages. There was nothing to be ashamed of and we were all here together. We all had the common ground.’ This exemplifies how inherent support of ethnically matched groups and facilitators is an important benefit to patient attitudes in tailored interventions.

Three quarters of studies in this review adapted the intervention to a suitable, convenient location for participants. Frequently reported barriers for engagement in health promoting behaviours in African-American women include unsafe neighbourhoods and lack of transportation. Community-based interventions using local, free community centres allows for increased accessibility, reduces effort to engage with the intervention and is perceived to achieve positive outcomes in self-management interventions. The study by D’Eramo Melkus *et al.* ^(22)^ demonstrated that increasing level of care points by including interventions in local pharmacies in under-served communities may have a beneficial impact over standard care. More evidence would be needed to support this claim as the study did not test for differences against standard care. Samuel-Hodge *et al.* ^(19)^ reported that effective community-based interventions can complement clinic-based care and lead to improved diabetes self-management. Only one study in this review included the use of both primary care and group sessions and found no significant improvement in glycaemic control^(25)^ but did find significant improvement in their primary outcome of physical activity levels.

This review included two studies with interventions held in church-based settings, both of which saw positive impacts on glycaemic control. A higher percentage of people of Black African ancestry are likely to characterise themselves as religious and attend religious services regularly compared with other groups^(57)^. As well as being a location of high point of contact, the churches play a major role in providing spiritual and social support for many attendees, providing them comfort to engage with trusted individuals. Therefore, using church-based settings enables the tailoring of interventions based on up to three domains (location, facilitator and messaging). The high attendance and comfort expressed in these venues makes churches ideal for reaching, recruiting and implementing self-management interventions, but the history of minorities being under-served and exploited can lead to suspiciousness and reluctance to participate that must be taken into consideration in public health implications^(58)^. It is evidenced that increasing point of access to care for these population groups along with having access to social support is beneficial for individual health outcomes in these population groups.

In this review, language tailoring was the least frequently used and included adjustments to literacy by use of simple teaching materials, assistance with forms and use of interactive and hands-on teaching methods. The sensitive use of language is largely seen as a surface structure of cultural tailoring with ability to increase comprehensibility and ultimately acceptability of the intervention. The integration of the language domain as a supplementary tailoring method may therefore have positive effects, but no studies in this review used it as a sole method of tailoring.

The messaging domain of Facilitator-Location-Language-Messaging integrates deeper structures of cultural tailoring by incorporation of cultural and social forces of ethnic communities. A majority of studies tailoring the messaging used diet as the method of tailoring where interventions tailored the nutritional curriculum to match pre-existing cultural beliefs. For example, in Lynch *et al.* ^(36)^, participants noted that categories of natural food were frequently labelled as God’s Food, so researchers used this pre-existing belief to build upon the nutritional curriculum to enhance participant understanding of macronutrient composition of foods and learning of new ways of eating. Fewer studies targeted faith (*n* 4) and only one study targeted family^(33)^. Interestingly, as well as finding a significant improvement in HbA1c, the use of family dyads also demonstrated improvements in health markers in the member of family that did not present with T2D. Certain family characteristics have been associated with poor diabetic outcomes^(59)^, suggesting that by encouraging family togetherness and interaction it may help to prevent diabetes onset. However, the current study was the only one using family and used a control receiving no treatment, thereby limiting evidence for the inclusion of family members and warranting further investigation for potential public health implication.

The strengths and limitations of our review warrant consideration. The focus on only RCT allowed for evaluation of differences between control and intervention groups in relation to culturally tailored components though it means other evidence was excluded that could have provided insights. All studies included in this systematic review were conducted in African-Americans in deprived communities of the USA. Due to the differences in culture and racial categorisations and the lack of sensitivity of these categories, it is unclear if these results can be successfully translated to other regions, including the UK. A further limitation is the very limited RCT in relation to prevention of T2D in populations of Black African ancestry.

In conclusion, this systematic review presents evidence on the effectiveness of culturally tailored interventions for diabetes management, with further evidence needed to support preventative implications. The evidence shows that the most common forms of tailoring were location and facilitator, with these showing the most success in improving glycaemic control. Interventions that tailored to more than one domain were, in general, more successful at improving glycaemic control in this population. Interestingly, these studies showed no association between weight loss and glycaemic control; however, the tailoring of interventions has led to increased knowledge and ultimately improvement in glycaemic control. Knowledge improvement is hence a potential driving force in the beneficial outcomes associated with tailored interventions. Heterogeneity in methods of tailoring, outcomes and control groups was evident across this review therefore limiting conclusions that can be drawn about the effectiveness of these interventions. The use of a standardised definition of cultural tailoring is needed to compare findings and for the creation of effective public health policy.

## Supplementary material

For supplementary material accompanying this paper visit https://doi.org/10.1017/S1368980021003682. click here to view supplementary material
