# Supplementary material for: Culturally tailored lifestyle interventions for the prevention and management of type 2 diabetes in adults of Black African ancestry: a systematic review of tailoring methods and their effectiveness
Source: Public Health Nutr. 2021 Aug 26;25(2):422–36. doi: 10.1017/S1368980021003682 (PMC8883766; doi:10.1017/S1368980021003682)
Supplement: Supplementary file 1 [file S1368980021003682sup.zip › S1368980021003682sup001.docx]

***Supplementary Material: MEDLINE Search Strategy***

1. exp African Continental Ancestry Group/

2. exp African Americans/

3. exp Minority Groups/

4. Education/

5. exp Educational Status/

6. exp Self Care/

7. dietary management.mp.

8. exp Self Efficacy/

9. exp Health Knowledge, Attitudes, Practice/

10. exp Health Promotion/

11. exp Life Style/

12. exp Rehabilitation/

13. exp Communication/

14. exp Social Support/

15. exp Patient Participation/

16. exp Patient Compliance/

17. exp Consumer Participation/

18. exp Counseling/

19. exp Community Mental Health Services/ or exp Community Health Services/ or exp Community Health Nursing/

20. exp Communication Barriers/

21. (complianc* or adherenc*).tw,ot.

22. (educat* or cultur* or instruct* or information* or program*).tw,ot.

23. (self adj6 (care or efficac* or group* or manag* or monitor*)).tw,ot.

24. (health knowledge* or rehabilitation* or communication*).tw,ot.

25. (life style or life?style).tw,ot.

26. counsel*.tw,ot.

27. (structured treatment* or teaching program*).tw,ot.

28. or/4-27

29. exp Diabetes Mellitus, Type 2/

30. (MODY or NIDDM or T2DM or T2D).tw,ot.

31. (non insulin$ depend$ or noninsulin$ depend$ or noninsulin?depend$ or non insulin?depend$).tw,ot.

32. ((typ? 2 or typ? II or typ?2 or typ?II) adj3 diabet$).tw,ot.

33. (((late or adult$ or matur$ or slow or stabl$) adj3 onset) and diabet$).tw,ot.

34. or/29-33

35. exp Diabetes Insipidus/

36. diabet$ insipidus.tw,ot

37. 35 or 36

38. 34 not 37

39. Hypertension/

40. hypertens$.tw.

41. (blood adj pressure).tw.

42. or/39-41

43. randomized controlled trial.pt.

44. controlled clinical trial.pt.

45. randomi?ed.ab.

46. placebo.ab.

47. drug therapy.fs.

48. randomly.ab.

49. trial.ab.

50. groups.ab.

51. or/43-50

52. exp Meta-Analysis/

53. exp Technology Assessment, Biomedical/

54. exp Meta-Analysis as Topic/

55. hta.tw,ot.

56. (health technology adj6 assessment$).tw,ot.

57. (meta analy$ or metaanaly$ or meta?analy$).tw,ot.

58. ((review$ or search$) adj10 (literature$ or medical database$ or medline or pubmed or embase or cochrane or cinahl or psycinfo or psyclit or healthstar or biosis or current content$ or systemat$)).tw,ot.

59. or/52-58

60. 38 or 42

61. or/1-3

62. 61 and 28 and 60

63. 62 and 59
